# Placing the values and preferences of people most affected by TB at the center of screening and testing: an approach for reaching the unreached

**DOI:** 10.1186/s44263-023-00027-0

**Published:** 2023-11-21

**Authors:** Andrew D. Kerkhoff, Nora S. West, Maria del Mar Castro, David Branigan, Devasahayam J. Christopher, Claudia M. Denkinger, Nguyen Viet Nhung, Grant Theron, William Worodria, Charles Yu, Monde Muyoyeta, Adithya Cattamanchi

**Affiliations:** 1 Division of HIV, Infectious Diseases and Global Medicine Zuckerberg San Francisco General Hospital and Trauma Center, University of California San Francisco, San Francisco, CA, USA.; 2 Center for Tuberculosis, University of California San Francisco, San Francisco, CA, USA.; 3 Pulmonary, Critical Care Allergy and Sleep Medicine, University of California San Francisco, San Francisco, USA.; 4 Division of Infectious Diseases and Tropical Medicine, Center of Infectious Diseases, Heidelberg University Hospital, Heidelberg, Germany.; 5 Treatment Action Group, New York, NY, USA.; 6 Department of Pulmonary Medicine, Christian Medical College, Vellore, India.; 7 German Center of Infection Research, Partner Site Heidelberg University Hospital, Heidelberg, Germany.; 8 University of Medicine and Pharmacy, Vietnam National University Hanoi, Hanoi, Vietnam.; 9 DSI-NRF Centre of Excellence for Biomedical Tuberculosis Research; South African Medical Research Council Centre for Tuberculosis Research; Division of Molecular Biology and Human Genetics, Faculty of Medicine and Health Sciences, Stellenbosch University, Cape Town, South Africa.; 10 Division of Pulmonology, Mulago National Referral Hospital, Kampala, Uganda.; 11 World Alliance for Lung and Intensive Care in Uganda, Kampala, Uganda.; 12 Center for Tuberculosis Research, De La Salle Medical and Health Sciences Institute, City of Dasmarinas, The Philippines.; 13 Centre for Infectious Disease Research in Zambia, Lusaka, Zambia.; 14 Division of Pulmonary Diseases and Critical Care Medicine, University of California Irvine, Irvine, CA, USA.

**Keywords:** TB, Preferences, Values, Patient centered, Diagnosis, Care engagement

## Abstract

To reach the millions of people with tuberculosis (TB) undiagnosed each year, there is an important need to provide people-centered screening and testing services. Despite people-centered care being a key pillar of the WHO END-TB Strategy, there have been few attempts to formally characterize and integrate the preferences of people *most* affected by TB — including those who have increased exposure to TB, limited access to services, and/or are at increased risk for TB — into new tools and strategies to improve screening and diagnosis. This perspective emphasizes the importance of preference research among people *most* affected by TB, provides an overview of qualitative preference exploration and quantitative preference elicitation research methods, and outlines how preferences can be applied to improve the acceptability, accessibility, and appropriateness of TB screening and testing services via four key opportunities. These include the following: (1) Defining the most preferred features of novel screening, triage, and diagnostic tools, (2) exploring and prioritizing setting-specific barriers and facilitators to screening and testing, (3) understanding what features of community- and facility-based strategies for improving TB detection and treatment are most valued, and (4) identifying the most relevant and resonant communication strategies to increase individual- and community-level awareness and demand. Preference research studies and translation of their findings into policy/guidance and operationalization have enormous potential to close the existing gaps in detection in high burden settings by enhancing the people-centeredness and reach of screening and diagnostic services to people *most* affected by TB who are currently being missed and left behind.

## Background

In 2021, more than 4 million people with tuberculosis (TB) remained undiagnosed or were not notified [1]. The diagnostic gap — representing people with TB who never access screening and testing services, whose diagnosis is missed despite accessing such services, or who accessed services and were diagnosed but not notified to the health system — globally accounts for the majority of individuals lost throughout the TB care cascade [1, 2]. Missed and delayed diagnosis is a key factor contributing to why TB remains a leading cause of death globally [1]. Finding more people with TB, and reaching them sooner, is essential for improving livelihoods and outcomes among people with TB and for interrupting transmission. Therefore, there is an urgent need to identify approaches that can inform the development and design of tools and strategies that can help close the large gaps in TB detection globally, by reaching people who are currently being missed and left behind by TB services.

## Importance of people-centered approaches for improving TB detection

The World Health Organization’s (WHO) END-TB Strategy serves as the global blueprint for TB care and prevention, and by 2030, it seeks to reduce TB incidence by 80%, TB mortality by 90%, and have 100% of TB-affected families protected against catastrophic costs [3, 4]. The first pillar of the END-TB Strategy is the provision of integrated, people-centered care and prevention, which includes systematic screening of most-at-risk groups and early TB diagnosis [4]. WHO defines a people-centered approach as “systematically assessing and addressing the needs, values and preferences of patients and providing educational, emotional and economic support to enable them to complete the diagnostic process and the full course of prescribed treatment” [5]. It is increasingly recognized that the meaningful incorporation of perspectives and preferences (or lack thereof) of people affected by TB — individuals with current or prior TB disease, their caregivers and immediate family members, and persons from key populations who are the *most* affected by TB (see below and Table 1) — is a key factor that can influence the reach and effectiveness of existing health services as well as new health interventions [6, 7]. People-centeredness of care also represents a distinct health outcome and is a critical metric for assessing the quality of health services [8, 9].

Encouragingly, an increasing number of TB programs in high burden settings have adopted people-centered approaches for providing TB services, although few have focused on screening and diagnosis [11, 12]. It is notable that to date, one key stakeholder’s priorities have often been missing from the development and design of TB products and services — that of people affected by TB. However, research to explore and characterize their values and preferences is essential to our ability to meet their needs and wants. In the following sections, we highlight the importance of including people *most* affected by TB in preference research, provide an overview of promising research methods for exploring and quantifying preferences, outline key opportunities for characterizing and incorporating TB-affected people’s perspectives and preferences into tools and strategies to improve TB detection, and discuss important considerations for conducting preference research studies in high TB burden settings.

## Focusing on the needs and wants of the people most affected by TB

There is an important need to reconceptualize people *most* affected by TB — who likely comprise the vast majority of the millions of people with TB who remain undiagnosed each year — as priority stakeholders in TB screening and testing activities and identify opportunities to understand their challenges, perspectives, and preferences through preference research studies. People most affected by TB infection and/or disease includes people who (1) have increased exposure to TB due to where they live and work, (2) have limited access to quality TB services, and (3) have an increased risk for TB due to biological or behavioral factors that compromise their immune system (Table 1) [10]. Though specific risk groups may differ from setting to setting, several key populations are especially vulnerable to TB and are most at risk for being left behind. Therefore, inclusion of these groups in future preference research should be prioritized, including the following: people living in poverty in both urban and rural settings, people who are contacts of people with TB, children, people living with HIV, miners, people who use substances (including illicit drugs, heavy alcohol use, and smoking), prisoners, migrants and refugees, indigenous populations, and healthcare workers [10]. In addition, future TB preference research should focus on including men, a group that is often overlooked despite accounting for a majority of global TB cases (including those never diagnosed) [1, 13, 14], facing an increased risk of TB disease and experiencing poorer outcomes largely due to gendered behaviors and risk factors [15–19]. Ultimately, insights gained from preference research among people *most* affected by TB will facilitate the development and implementation of TB tools and strategies that are more acceptable, accessible, and appropriate and that therefore have the potential for greater reach, equity, and public health impact.

## Methods for exploring and quantifying peoples’ preferences for TB screening and diagnosis

A systematic review of the literature identified more than 30 unique preference research methods, including 10 qualitative methods for exploring health-related preferences (“preference exploration methods”) and 22 quantitative methods for estimating the value, importance, or desirability of health-related features and outcomes (“preference elicitation methods”) [20]. While each of these methods have intrinsic strengths and weaknesses, Table 2 provides a brief overview of preference exploration and elicitation methods that we believe may have the greatest utility (considering potential data outputs and insights generated) and feasibility (considering resources required and complexity) for application in resource-limited, high TB burden settings. Of note, men and women likely face distinct barriers to TB diagnosis and care across diverse contexts and have unique healthcare-related preferences [14, 21–24]; thus, whenever possible, incorporating a gender lens into research applying any of the methods outlined below is crucial to more thoroughly understanding the perspectives, values, and needs of both groups.

In-depth interviews (including unstructured and semistructured) and focus-group discussions are the most flexible and promising available qualitative methods for exploring peoples’ priorities and preferences at all stages of medical and public health research [25–29]. In-depth interviews, which consist of open-ended questions, allow for collection of rich and detailed data on an individual’s choices, feelings, and lived reality. Focus groups, where a relatively homogenous group of people based on a characteristic of interest (e.g., miners or household members of people with TB) are formed for discussion of a topic using open-ended questions, are a more economical approach to qualitative data collection [25–29]. Focus-group formats are used to stimulate thinking, engender comfort discussing difficult topics, and understand reasons for group consensus or disagreement. In-depth interviews and focus-group discussions use purposive sampling to select participants based on characteristics of interest, which provides a unique opportunity to gain perspectives from both individuals who engage in a behavior, health service, or other activity of interest and, importantly, those who do not. The flexibility of purposive sampling and qualitative data collection can provide detailed and rich preference perspectives from those most affected by TB in high burden settings.

Among available preference elicitation methods, best-worst scaling (BWS) [32–34] and discrete choice experiments (DCE) [35–37] embedded within surveys among individuals are highly promising and robust methods for quantifying the relative value or importance of all types of attributes (e.g., features, characteristics, statements, outcomes, other items) and their respective levels (i.e., different forms attributes can take). Both BWS and DCEs are grounded in human choice behavior theory (i.e., random utility theory) [41, 42] and determine the strength of people’s preference through a series of questions, called choice tasks. They are increasingly being utilized in global health research [43, 44] due to their broad applicability for answering many types of research questions among different stakeholders [37], their ability to quantify the trade-offs people are willing to make to have their most preferred features, and in part due to the availability of end-to-end software solutions (i.e., support design, implementation, and analysis) that make them more accessible. One major benefit of both DCEs and BWS is their ability to characterize preference heterogeneity in a population through latent class analysis (LCA) [45, 46]; LCA not only can identify groups of persons with similar, unique preferences that might otherwise be missed when undertaking sub-group analysis (e.g., by age, sex, HIV status, or prior TB disease) but can also estimate the relative size of such groups (also known as “preference archetypes”). Knowledge of preference archetypes can help to determine whether TB programs may need to provide different testing options or have tailored components of case finding or communications strategies that reach and appeal to different people affected by TB (see key opportunities no. 1, no. 3, and no. 4 below). Undoubtedly, there are important lessons that can be learned from HIV programs in resource-limited settings with respect to HIV self-testing strategies and differentiated service delivery models informed by preference research that could be extended to and adapted for TB [47–52].

However, both BWS and DCEs are somewhat more complex than alternative methods and may not be feasible to undertake depending on available resources and expertise. In settings and situations where less rigor is needed, and/or less complex designs are required for quantifying preferences and values, survey-based preference assessments, including allocation of points, as well as ranking and ratings questions can still provide efficient and important insights into people’s preferences and values [30, 31]. However, it is important to be aware of their potential limitations (Table 2). Ultimately, the combined use of qualitative and quantitative preference methods, when possible, will provide the most powerful insights into people’s perspectives and preferences by not only elucidating what factors are the most important or acceptable but also understanding the reasons and context that underpin those perspectives and preferences.

## Key opportunity no. 1 — Informing test development: defining the most preferred features for novel TB screening, triage, and diagnostic tools

To close existing gaps in TB detection, it is important to start by understanding which features of TB screening, triage, and diagnostic tools and approaches (henceforth “TB tests”) are the most important to people affected by TB and are likely to appeal to them. Currently, the development of new TB tests is guided by WHO target product profiles (TPPs) [53]; these address key priorities of TB test development that were informed by the perspectives and recommendations of healthcare providers, researchers, product developers, and policy officials, but not the people who are likely to undergo or have undergone TB testing in high burden settings.

In-depth interviews among people affected by TB can garner insights into the importance of different features of tests (Table 3), what trade-offs may or may not be acceptable (e.g., convenience of decentralized, community-based testing and rapid results for lower accuracy), what drives those attitudes and preferences, and how the availability of preferred test features may or may not motivate and facilitate improved health-seeking behaviors and potentially earlier diagnosis [54]. Furthermore, quantitative techniques, especially DCEs and BWS, can complement qualitative approaches by determining the relative importance of different test attributes (e.g., test accuracy is twice as valued as the location where testing is performed), quantify acceptable trade-offs (e.g., the average person would accept 10% lower sensitivity if same-day results were available), and can even simulate an individual’s predicted choice of different novel tests (in the context of available tools) if they were to be implemented. Mixed-methods preference research will be especially important for understanding perspectives on next-generation TB tests that can be performed in community settings (or one day even at home) and/or that utilize non-sputum-based samples, to characterize not only the potential demand for such tests but also the potential concerns (e.g., less trust in results, lack of self-efficacy for self-testing, less acceptable sample type).

The results of preference research can help product developers design tests that are more likely to be used by health workers and demanded by people seeking TB care. In particular, the direct incorporation of preference evidence into revised and updated TPPs is critical to ensure that product developers focus on tests that people affected by TB and their health workers find to be the most acceptable and appealing. In addition to informing the development of future TB tests, preference research studies should also be undertaken in parallel with diagnostic accuracy assessments of late-stage TB tests to generate key evidence for policy decisions that will determine whether national and international decision-makers recommend their use [55, 56].

## Key opportunity no. 2 — Identify existing barriers: exploring and prioritizing barriers and facilitators to TB screening and testing services

Across different settings, people with undiagnosed TB may face many complex barriers at each step of the care pathway [21–23, 57]; to be diagnosed with TB, an individual must potentially overcome barriers to healthcare seeking after symptom onset, barriers to physically accessing TB screening and diagnostic services, and barriers to having a diagnosis made after accessing services. Therefore, to design community- and facility-based strategies that can improve TB diagnosis and care engagement by being responsive to people’s needs, it is crucial to understand the determinants that influence the ability and likelihood of people with undiagnosed TB to receive a TB diagnosis. In-depth interviews and focus-group discussions with TB-affected individuals should seek to explore the range of barriers and facilitators they did face (if they have current/prior TB disease) or may face (if they are at risk) along their pathway to TB care in the context of their daily lived reality; special attention should be paid to individuals with divergent experiences, perspectives, and preferences, as these so-called “edge cases” may be people who are the least likely to access TB screening and diagnostic services. Additional evidence can be generated about barriers and facilitators to TB screening and diagnosis through a review of relevant qualitative studies. Systematic reviews and meta-syntheses of qualitative literature have been undertaken to synthesize evidence on a variety of topics that can inform preference research, including TB in migrant populations, uptake of TB diagnostic and treatment services in hard-to-reach populations, and gender-related barriers and delays to TB screening, diagnostics, and treatment [22, 54, 58]. Qualitative preference data can further inform quantitative preference research methods that can help to either rank or determine the relative importance (e.g., BWS or allocation of points) of individual’s barriers to TB diagnosis [59]. Since no case finding or communications strategy can target all relevant barriers, these insights are important to identifying which barriers should be prioritized and which facilitators should be leveraged. Similar approaches can be applied to understand and prioritize barriers to linkage to TB treatment after individuals are reached by community-based case finding strategies, including through household contact tracing.

**Fig. 1:**
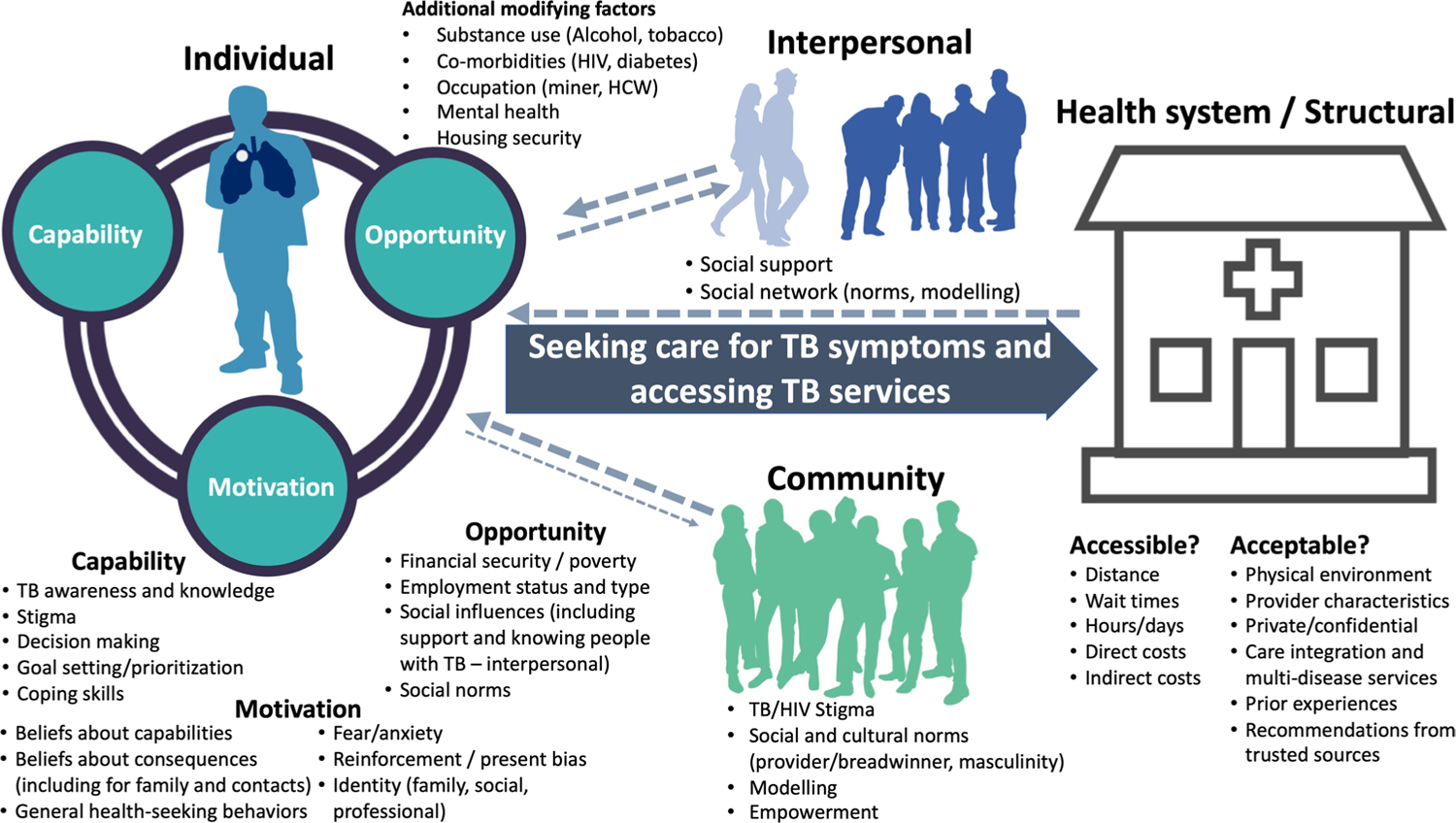
Conceptual model of people most affected by TB’s potential barriers to healthcare seeking and accessing screening and diagnostic services to be explored and assessed using preference research methods. Barriers are characterized according to the capability, opportunity, and motivation behavior change model (COM-B) and Theoretical Domains Framework (TDF) [60, 61]. An understanding of contextually relevant barriers is crucial for designing preference-informed TB detection and communication strategies that overcome such barriers to improve TB diagnosis and care engagement among people most affected by TB. While this figure focuses on barriers to care seeking and accessing TB services for people at risk for TB, it is important to note that such individuals also face barriers to diagnosis after accessing services, including the limited capability of health services to identify those at risk for TB and to provide appropriate screening and/or diagnostic testing

To increase the likelihood that all relevant barriers to TB screening and diagnostic services are identified and to guide subsequent intervention and strategy development by programs and researchers, preference research should be grounded in individual-level behavior change theories, such as the COM-B/theoretical domains framework (TDF) (Fig. 1) [60, 61]. COM-B/TDF posits that to change behavior (e.g., improve care seeking or accessing TB services), an individual’s capability, opportunity, and/or motivation must be positively shifted; the application of COM-B/TDF allows for individual-level barriers to and facilitators to behavior change to be systematically assessed and categorized. While designing community- and facility-based strategies to improve TB detection, it is important to consider which mechanisms are most likely to facilitate individuals’ engagement into TB services. To do so, individuals’ multi-level, key barriers can be directly linked to behavior change techniques (BCTs) that are most likely to overcome such barriers as part of a stepwise intervention or strategy design approach (e.g., using the behavior change wheel or intervention mapping) [60, 62–64]. In addition to individual-level behavior change theories, frameworks such as WHO’s Conceptual Social Determinants of Health Framework [65], which presents the interplay between socioeconomic and political setting, structural and social determinants, and health inequity, may be important to use when both assessing barriers and facilitators to the TB diagnostic process and in addressing these through multicomponent strategies.

## Key opportunity no. 3 — Informing TB detection strategies: understanding what features of strategies to improve TB care engagement are most preferred

Once setting-specific barriers to accessing TB screening and diagnosis services are known and have been prioritized, TB case finding, and quality improvement strategies, must strive to directly address these barriers. Ultimately, this will require improvements to facility-based services to become more accessible and acceptable, as well as the implementation of community-based strategies that provide convenience, flexibility, and choice to those who may not be able to or want to access traditional facility-based health services [66, 67]. There are many potential design and delivery considerations for TB case finding and TB diagnostic service improvement strategies; preference research methods have a key role in helping to elucidate which features and components are most preferred or important and may therefore be the most likely to overcome present barriers and improve diagnosis and care engagement (Table 3). Both in-depth interviews and focus-group discussions among people affected by TB can not only to explore specific features or modes of delivery that may be preferred but also to understand why they are or are not valued. A study utilizing both focus groups and in-depth interviews among persons with TB, household members of persons with TB, and health workers in South Africa found multifaceted reasons why household visits to screen for TB among individuals at risk may or may not be preferable, including trade-offs between convenience and economics factors (e.g., transport and wages lost to seek testing), and the factors that influence perceived likelihood of stigmatization [68]. Preference elicitation methods can then help to determine which features or options are the most acceptable or appealing and, in the case of DCEs, can also give important insights into how different combinations of strategy features or delivery options are expected to strengthen or weaken individual’s preferences. Upon completion, the findings garnered from preference research studies should then directly inform the design of people-centered strategies to improve TB diagnosis as part of a stakeholder-engaged, theory-informed, step-wise process (see “Key opportunity no. 2 — Identify existing barriers: exploring and prioritizing barriers and facilitators to TB screening and testing services”).

As an example, a DCE among persons with TB in Zambia found the strongest preferences for the addition of same-day TB test results as a strategy to improve existing TB diagnostic services, and that services would be even more appealing when same-day results were combined with either enhanced privacy and confidentiality, or a small testing-conditional financial incentive [69]. These preferences were most pronounced among individuals who reported prolonged delays in seeking care for their TB illness, suggesting that the implementation of same-day test results and other preferred strategies may improve TB diagnosis in this setting by overcoming existing barriers and accelerating care engagement.

## Key opportunity no. 4 — Improving TB communication: identifying the most relevant and resonant communication strategies to increase individual- and community-level awareness and generate demand for TB services

To reach more persons with TB and diagnose them sooner, it will not be enough to only “push” out new tools and strategies — demand must be generated through “pull” strategies that include tailored communications that not only increase TB-related knowledge and awareness but also motivate action among individuals to actively seek out TB diagnostic screening and testing services [70]; communications to increase awareness and generate demand for TB services should complement any TB case-finding strategy. Preference research methods represent important tools for determining aspects underpinning an effective communication strategy that people most affected by TB value the most: (1) what are their most preferred and trusted channels for accessing and receiving health information (e.g., TV, radio, social media, posters/billboards, newspaper, pamphlets, SMS, community dramas, face to face); (2) what are their most preferred and trusted messengers (e.g., healthcare workers, family members, peers, religious leaders, practitioners of traditional, alternative, or complementary medicine, and other community and national leaders, celebrities); (3) what specific messages are the most resonant and appealing (e.g., accentuating the benefits of early TB diagnosis or emphasizing the risks of delayed TB diagnosis); and (4) what non-message-related features of media-based communication strategies (i.e., broadcast, digital, and print) are the most resonant and appealing (e.g., images, colors) (Table 3). There may also be a particular need in many settings to determine communications preferences related to how to best address and overcome TB-related stigma [22, 54, 71].

In settings where these preferences are relatively unknown, especially for people *most* affected by TB, qualitative methods are an important first step for exploring different dimensions of communications-related preferences, especially to understand how they may relate to and could potentially modify key barriers to accessing TB services (see “Key opportunity no. 2 — Identify existing barriers: exploring and prioritizing barriers and facilitators to TB screening and testing services” above). They may also explore alternatives to reach populations with a diversity of languages and traditions, such as indigenous people and migrants, and identify key gatekeepers within these populations who may need to be engaged for communication messages to be appropriately developed and disseminated. Quantitatively, BWS has been used in commercial marketing to test which marketing claims (statements about the benefits or performance of a products or service) are the most appealing to target consumers, and the results are used to increase awareness of their product and persuade and motivate consumers to purchase their product [32]. This suggests that BWS may be well-suited for determining which channels, messengers, and messages should be prioritized for incorporation into an awareness raising and demand generation campaign given its ability to quantify the relative importance of large number items. Ultimately, the application of preference research methods will help to ensure that TB-related communications are more accessible, understandable, trusted, relevant, and resonant to target populations and achieve their objective of increasing TB awareness and uptake of TB screening and diagnostic services [72]. To maximize reach, it is important that communication strategies also be informed by and advanced in partnership with in-country civil society organizations and advocates that regularly engage communities and people affected by TB.

In addition to helping create demand for TB screening and diagnostic services, preference research also has the potential to ensure that communication of TB test results is aligned people’s values and preferences. For example, a survey of household contacts in Uganda revealed that while access to mobile phones was nearly universal, almost half preferred to receive the detailed results of their test in person rather than via SMS [73]. Similarly, a DCE among TB patients in Zambia found they had very strong negative preferences receiving their test results by SMS, and that they would rather return to the facility in-person to learn their results [69]. In both cases, this demonstrates that a well-intentioned, convenient intervention could undermine people-centeredness and possibly care engagement due to concerns related to privacy and/or stigma.

## Weighing trade-offs between the preferences of different stakeholders

Ideally, the features of TB tests, case finding strategies, and communications strategies that TB-affected people most strongly prefer would be prioritized for implementation — however, this must also be balanced against the preferences and perspectives of other key stakeholders as well as available resources. When possible, preference research should be undertaken among different stakeholders, especially health workers who provide TB services and local/national decision-makers who influence TB-related policy. Among health workers, preference research should explore their current realities, including workloads and expectations [74, 75], and assess perceived acceptability, feasibility, and preferences for novel TB tests and potential features of new TB detection approaches and strategies (considering characteristics of the innovation such as its strength of evidence, relative advantage, adaptability, trialability, complexity, and design [6, 76]). Preference research among decision-makers should seek to determine the perceived importance of initiatives to improve TB detection relative to other TB-related and public health priorities. This involves discerning what factors — such as impact, cost, equity, and available alternatives — may drive them to fund and support the implementation of new TB tests and case finding strategies. To this point, once preference data from different stakeholders is available, further data may be needed by decision-makers to understand the costs and cost-effectiveness of stakeholders’ more preferred and less preferred (and potentially lower cost) tools and strategies.

Historically, decision-making bodies such as National TB Programs and the WHO Global TB Program have heavily based recommendations on efficacy-/effectiveness-focused evidence garnered from well-conducted studies, including diagnostic accuracy evaluations and individual and cluster randomized controlled trials (RCTs). The evidence-to-decision (EtD) framework goes beyond efficacy/effectiveness alone and provides a systematic and holistic way for weighing the values and attitudes of all stakeholders in the context of all other evidence (benefits and harms, resources required, cost-effectiveness, equity considerations, acceptability, and feasibility) [55, 56]. Applying the EtD framework can help decision-makers at all levels account for preferences and determine whether specific TB tests, case finding strategies, and communications strategies should be recommended and/or implemented. These approaches can help to ensure contextual relevance and appropriateness and will increase the likelihood that preference-informed, people-centered TB tests and diagnostic strategies are adopted, implemented, and sustained.

## Practical considerations and challenges for conducting preference research studies in high burden settings

There are many considerations and potential challenges associated with the conduct of preference research in high TB burden settings. Currently, most TB screening and diagnostic research are conducted among people with presumed or confirmed TB recruited from health facilities for understandable, pragmatic reasons; however, people who do not seek or who are unable to access TB care (and are representative of the millions of people with TB who remain undiagnosed each year) likely have differential and unique barriers, perspectives, and values. Thus, one of most important considerations for future preference research is how to access and include people most affected by TB who are not being reached by TB services. Approaches may include the following: (a) recruiting at community-based locations, venues, and events (e.g., markets, bars, community halls, churches, minibus stands) or from among household and other close, non-household contacts not yet engaged in care, (b) the use of snowball sampling, and (c) partnering with trusted non-healthcare figures (e.g., religious leaders, traditional healers, champions) and local advocacy groups to support recruitment efforts.

Notably, there is limited guidance available for selecting the most appropriate preference research method(s), and we do not advocate for one specific approach over another as they have differing strengths, limitations, and potential complementariness (see Table 2). The methodologic approach (and subsequent design) should be determined in collaboration with research and implementing partners with consideration for the following: (a) the overall goals of the research (exploration or elicitation [or both], need for assessment of trade-offs, or characterization of preference heterogeneity); (b) what resources are available (time, funding, personnel, methodologic experience/expertise); and (c) the characteristics of potential participants (age [potential cognition], education/literacy, possible language, or cultural barriers) [77]. It is also worth highlighting that the lack of local experience or available expertise should not necessarily preclude undertaking the most appropriate preference studies — their design, implementation, and analysis, provide important opportunities for partnership to facilitate knowledge sharing, and to develop capacity in these important and versatile methods.

An additional challenge of some preference research is that it may sometimes include hypothetical options (e.g., that are not yet available in a setting or do not exist), which can create bias toward known features, as it can be hard to know how much one may like or dislike something if they have never had the opportunity to experience it [78]; use of standardized descriptions with simple language, combined with pictures, videos, and/or props, can be helpful in these situations, but may not be able to eliminate hypothetical bias altogether. Furthermore, in some settings, preference research participants may not be used to or traditionally be allowed to share their perspectives, preferences, and values; in such cases, qualitative methods may be especially powerful for not only preliminary exploration of preferences and concerns/challenges related to sharing their perspectives but also to understand the potential feasibility and appropriateness of different preference elicitation methods. For both qualitative and quantitative preference research studies among people affected by TB, it is important to develop procedures that encourage their full and honest participation; to facilitate this, community advisory boards (CABs) and other civil society advocacy groups in local settings can be engaged to advise on research study design and procedures (e.g., the use of culturally appropriate and empowering language emphasizing the importance of their perspectives) [79].

A final key challenge involves operationalizing the findings from preference research among TB-affected individuals within the constraints of fixed, often under-resourced TB programs, especially when substantial preference heterogeneity is present. Yet HIV programs, operating in the same resource-limited settings as TB programs, support that realizing this people-centered approach is feasible. They offer vital lessons for scaling up and sustaining strategies that prioritize providing individuals choice beyond singular, facility-based options (e.g., modality, decentralized access points, different forms of support, availability of additional services) at each step of the care continuum to enhance client satisfaction and engagement and retention in care [52, 80]. Therefore, while the provision of choice and a people-centered approach within TB diagnostic and screening programs to meet the diverse needs of TB-affected individuals is challenging, it is possible, with cultivating and sustaining political will, and a reimagined sense of what is considered “feasible” in the current landscape of TB programs.

## Conclusions

Reaching the millions of individuals in high burden settings with undiagnosed TB will require novel approaches, tools, and strategies combined with multi-sectoral partnerships, strong political will, and sustained funding. Preference research methods encompass both qualitative and quantitative techniques that can explore and quantify the strength of TB-affected peoples’ preferences toward an improved understanding of their perspectives, including relevant barriers, what may or may not be acceptable, and, ultimately, what they value most. The increased application of preference research methods among people affected by TB represents one highly promising approach for closing existing gaps in TB detection by prioritizing the development and implementation of preference-informed, people-centered TB tests, case finding, and communication strategies that are responsive to their needs and wants.

## Figures and Tables

**Table 1:** Overview of people most affected by TB to prioritize for inclusion in TB screening and diagnosis preference research (table adapted from STOP TB Partnership [10]

People who have **increased exposure** to TB due to where they **live or work**	**People who live**	**People who work**
	In overcrowded environments In poorly ventilated or dusty conditions In urban slums	In overcrowded environments In poorly ventilated or dusty conditions In health care settings
	**These are:** incarcerated persons, persons in long-term care facilities, sex workers, miners, healthcare workers, community health workers, household members of persons with TB	

People who have **limited access** to TB services	**People who live**	**People who are**	**People who have**
	In hard-to-reach areas	From tribal populations or indigenous groups Homeless Lesbian, gay, bisexual or transgender	Mental illness, developmental or physical disabilities Legal barriers to accessing care
	**These are:** migrant workers, women in settings with gender disparity, children, refugees or internally displaced persons, illegal miners, and undocumented migrants		
People at **increased risk** for TB due to **behavioral or biological factors** that impact immune function	**People who live**	**People who are**	**People who**
	With HIV With diabetes With silicosis	Undernourished Undergoing immunosuppression therapy	Use tobacco Inject drugs Suffer from alcohol use disorders

**Table 2:** Overview of recommended preference research methods for use in high TB burden resource-limited settings

Method		Description		Strengths		Potential challenges
***Qualitative preference methods (preference exploration)***						
**Focus-group discussions (FGDs)** [25–29]		Focus groups serve multiple purposes: they can stand alone as a valuable source of group and individual insight or can enhance understanding of findings from quantitative methods like surveys. In focus groups, participants discuss a topic in response to a short set of focused questions, usually ranging from 4 to 10 questions total. A moderator leads the conversation, ensuring that it stays on topic and managing discussion dynamics, while a notetaker captures non-verbal cues like facial expressions and body language. Focus groups excel in capturing diverse opinions from individuals who share certain characteristics, offering a rich understanding of preferences and viewpoints.		*Resources available*: There are many existing, accessible resources for developing FGD guides *Efficient data collection*: Provide an economical way to collect data that captures a wide range of responses *Provides rich insights*: Offers detailed explanations for individual and collective preferences, behaviors, and decision-making *Stimulates group thinking*: Participant comments stimulate the thinking of others, especially in the context of something preferred versus not preferred *Visual and prototype friendly*: FGDs are well-suited for the use of visual aids or prototypes to enhance discussions *Can provide instant reactions*: Allows for the observation and documentation of instant reactions to ideas or options, including nonverbal cues		*Depends on moderator skill*: The quality of FGD data relies on the moderator’s skill in guiding the group *Logistical challenges*: FGDs may encounter issues such as convening a group at the same time and securing an appropriate space *Confidentiality risk*: Confidentiality cannot always be ensured in a group setting *Negative group dynamics*: Dominant participants and/or power differentials may affect the expression of marginalized or “less acceptable” views *Potentially limited transferability*: Findings may not easily apply to other populations or settings *Requires time and expertise*: Transcription, translation, and analysis of FGD data can be time-consuming and requires expertise, particularly when dealing with complex data and study objectives
**Individual in-depth interviews (IDIs — semi-structured and unstructured)** [25–29]		IDIs are a powerful tool in health research to explore individual viewpoints. These interviews come in two types: semi-structured and unstructured. In semi-structured interviews, an interviewer follows a guide to steer the conversation. This guide includes key topics and open-ended questions to encourage thoughtful responses. Unstructured interviews have a general theme or topic of interest but do not follow a set list of questions, allowing for a more free-flowing discussion. Both approaches are excellent for gaining detailed reasons behind a participant’s preferences and choices.		*Resources available*: There are many existing, accessible resources for developing IDI guides *Promotes participant openness*: Encourages participants to freely share their insights and experiences in their own words *Enables flexible exploration*: Allows for the collection of in-depth information beyond what was initially planned as concepts or relevant topics arise during data collection *Provides rich insights*: Offers detailed explanations for individual preferences, behaviors, and decision-making *Private and comfortable*: The one-on-one nature fosters a private and comfortable setting, which may improve trust and sharing		*Depends on interviewer skill*: The quality of IDI data relies on the skill and judgment of the interviewer *Potential data comparison challenges*: Comparing data between different respondents can be difficult due to lack of standardization and varying responses *Time-intensive*: Conducting IDIs with each participant can be time-consuming for both participants and data collection teams *Potentially limited transferability*: Findings may not easily apply to other populations or settings *Requires time and expertise*: Transcription, translation, and analysis of IDI data can be time-consuming and requires expertise, particularly when dealing-with complex data and study objectives
***Quantitative preference methods (preference elicitation)***						
**Allocation of points questions** [30,31]	Allocation of points questions ask people to prioritize different features or attributes by giving them a set number of points, usually totaling 100. Participants distribute these points across the attributes, giving more points to what they value most and fewer to what they value less. They can even give zero points to attributes they do not find important. These questions are often part of a structured survey but can also be included in in-depth interviews for a more comprehensive mixed-methods approach.		*Simple to design*: Creating the questions is straightforward *Easy to administer*: Questions can be completed quickly and easily by most participants *Suitable for many contexts*: Can be used in a wide range of settings, including those where paper-based methods are required *Improved precision for preference estimates*: Provides more granular insight into preference differences between items or attributes compared to ranking and rating questions, but less than DCEs and BWS type 1 *Can identify unimportant attributes*: Participants can clearly indicate item attributes they deem unimportant by allocating zero points to them *Enables equal preference indication*: Participants can denote a tie by assigning identical point values to the various attributes they equally prefer *Simple analysis*: The data generated are easy to analyze and interpret		*Risk of framing effects*: The framing of the task can shape responses, necessitating careful consideration for how they are presented *Potential for higher cognitive burden*: Allocation and assigning precise values may be challenging and more mentally taxing than ranking or ratings questions and may affect response quality *May be confusing and difficult*: The process of translating preferences into a precise allocation of points could be confusing for some participants, especially for those with lower education *Limitation with paper-based methods*: When only paper-based methods are available, completing the allocation of points can be more difficult as the total number of points remaining is not continuously updated *Potential for unreliable data with many attributes*: With a large number of attributes (more than 5–7), it becomes challenging for participants to provide consistent rankings, which may affect the robustness of the data	
**Best-worst scaling (BWS) type 1** [32–34]	BWS type 1 is a type of choice experiment that is used to find out which items (statements, features, criteria, outcomes, etc.) people value or like the most and least. While there are three variations of BWS, type 1 (object case) is the most versatile, commonly utilized, and likely to be the most applicable for use in resource-limited settings. Participants engage in a series of questions, referred to as “choice tasks,” to reveal their preferences. In each of these tasks, they are usually shown a set of 4 to 5 different items and are asked to pick both their top favorite (best) and least favorite (worst).		*Clear and intuitive*: The task of selecting the best and worst from a set in each question is generally easy for most people to understand *Suitable for many items*: Can effectively assess a large number of items or attributes, making it a good choice when many must be evaluated *Theory based*: Grounded in random utility theory, which models real-world decision-making processes *High precision for preference estimates*: Provides precise estimates of the relative importance and magnitude of difference in preference between items *Can identify hidden preference groups*: Latent-class analyses can unearth “hidden” groups with similar preferences		*Risk of framing effects*: The framing of hypothetical choice scenarios can substantially shape responses, necessitating careful consideration for how choice tasks are presented *Large sample sizes often needed*: BWS type 1 typically requires larger sample sizes compared to other methods to ensure reliable and robust findings *Potential for high cognitive burden*: Making multiple best-worst trade-offs can be mentally taxing for participants, which may affect response quality; however, it may be more suitable for those with low health literacy than DCEs *Cannot determine absolute importance*: It does not allow for understanding of the overall importance of the attributes, including whether all, some, or none of the attributes is important to participants, without adding “anchoring” questions *Expertise and software required*: Both the design and analysis stages are simpler than DCEs but still require expertise and the utilization of statistical or specialized software	
**Discrete choice experiments (DCE)** [35–37]	DCE is a type of choice experiment used to find out what features or characteristics (i.e., attributes and attribute levels) people care about the most when given different options (i.e., profiles) that mimic products, services, or policies. Participants answer a series of questions, known as “choice tasks,” which help understand what they prefer. In each task, they usually see 2 or 3 different options that have differing features and pick the one they like the most. Some versions of DCEs allow participants to pick “none” if they do not like any options or to say if they would actually want or use their favorite option in real life if it were available.		*Mimics real-world decision making*: DCEs require participants to make trade-offs between options with different features, mirroring the decisions people commonly face in real-life situations *Theory based*: Grounded in random utility theory, which models real-world decision-making processes *High precision for preference estimates*: Provides precise estimates of the relative importance and magnitude of difference in preference between attributes and attribute levels *Can determine willingness to trade*: Enables assessment of the extent to which participants are willing to compromise on less preferred features for more preferred ones *Can identify hidden preference groups*: Latent-class analyses can unearth “hidden” groups with similar preferences *Simulation capabilities*: DCEs support simulations, which extrapolate participant preference data to predict real-world demand and uptake of different potential options		*Needs careful selection of attributes*: The development of the different attributes and their levels requires thoughtful consideration to ensure that key drivers of preference and decision-making are accounted for *Risk of framing effects*: The framing of hypothetical choice scenarios can substantially shape responses, necessitating careful consideration for how choice tasks are presented *Large sample sizes often needed*: DCEs typically require larger sample sizes compared to other methods to ensure reliable and robust findings *Potential for high cognitive burden*: Making multiple trade-offs can be mentally taxing for participants, especially those with low health literacy, which may affect response quality *Potential for unreliable data with many attributes*: With a large number of attributes (more than 5–7), it becomes challenging for participants to evaluate and choose options carefully, which may affect the robustness of the data *Expertise and software required*: Both the design and analysis stages of DCEs require a high level of expertise and the utilization of statistical or specialized software	
**Ranking questions** [30, 31]	Ranking questions ask participants to put items or attributes in order based on their personal preferences, according to how important or how desirable they are. These questions can take different forms: either as a paired comparison, where participants compare two items at a time and select the preferred option from the pair, or as a rank order, where participants list multiple items or attributes from the one they like most to the one they like least (or vice versa). Ranking questions are typically part of a structured survey but can also be included with in-depth interviews as part of a mixed-method approach.		*Simple to design*: Creating the questions is straightforward *Suitable for many contexts:* Can be used in a wide range of settings, including those where paper-based methods are required *Clear and intuitive*: The questions are generally easy for most people to understand *Easy to administer*: Questions can be completed quickly and easily by most participants *Quick relative importance assessment*: Efficiently identifies the relative importance of different items or attributes *Simple analysis*: The data generated are easy to analyze and interpret		*Risk of framing effects*: The way questions are framed (e.g., rank items best to worst or vice versa) can influence the responses, potentially leading to different rank orders. *Cannot determine preference magnitude*: It does not allow for understanding how much more one attribute is preferred over another, including possible ties (unless ties are explicitly allowed) *Cannot determine absolute importance*: It does not allow for understanding of the overall importance of the attributes, including whether all, some, or none of the attributes is important to participants *Potential for unreliable data with many attributes*: With a large number of attributes (more than 5–7), it becomes challenging for participants to provide consistent rankings, which may affect the robustness of the data	
**Rating questions** [30, 31]	Rating questions ask participants to give a score to show how much they prefer, value, or are satisfied with different items or attributes. These questions can use various types of scales, including the following: numerical (e.g., 1 to 5), Likert (e.g., from “strongly disagree” to “agree”), visual analogue (e.g., marking a point on a continuous line), semantic differential (e.g., from “bad” to “good”), and faces (e.g., from a sad to a happy face). Rating questions are usually part of a structured survey but can also be included with in-depth interviews as part of a mixed-method approach.		*Simple to design*: Creating the questions is straightforward *Suitable for many contexts:* Can be used in a wide range of settings, including those where paper-based methods are required *Clear and intuitive*: The questions are generally easy for most people to understand *Easy to administer*: Questions can be completed quickly and easily by most participants *Simple analysis*: The data generated are easy to analyze and interpret		*Risk of “yeah-saying” bias*: There is a chance participants may give answers they think are socially acceptable or to avoid conflict *Prone to “satisficing”*: Some participants might rush through by consistently choosing only the best, middle, or worst options on the scale *Variable scale interpretation*: The way people understand the scales can differ across cultures or settings, making it potentially hard to compare results *Limited discrimination between preferences*: Since no trade-offs are involved, people might rate many items or attributes as important, making it hard to distinguish between them	
***Mixed preference methods (qualitative and quantitative)***						
**Q-methodology** [38–40]	Q-methodology is a way to understand people’s different opinions or preferences for a given topic. First, participants go through a step called “Q-sorting,” where they rank items or attributes from least liked, important, or agreed with (negative values) to the most liked, important, or agreed with (positive values) using a special chart (i.e., “Q-sort grid”). Though optional, many participants are also interviewed afterward to dive deeper into why they made their choices. The information from the Q-sorting is then analyzed to understand what preferences or viewpoints exist and group people based on similar or different preferences and opinions.		*Mixes qualitative and quantitative data*: Combines the quantitative analysis of how people rank and sort items with a detailed understanding of why they do so *Suitable for many items*: Can effectively assess a large number of items or attributes, making it a good choice when many must be evaluated *Reveals diverse viewpoints*: With a well-selected group of participants, this method can reveal a wide range of opinions and preferences, showing areas of agreement and disagreement *Promotes deep thinking*: The engaging process helps people think about what they prefer or agree with		*Needs careful selection of attributes*: The development of the different items or attributes evaluated in the study requires thoughtful consideration *May be challenging for participants*: The sorting process may be hard for some, and adding more items or attributes can make it even more complex *Overall process may be time-consuming*: The multiple steps in both gathering and analyzing the data can require a substantial time commitment *Potentially complex analysis*: Understanding the results is complicated and requires someone with expertise in applying the method	

**Table 3:** Attributes and features related to TB tests, screening and diagnostic strategies, and communication strategies that can be evaluated through preference research methods

New tools	New case finding strategies		New communications strategies
Screening, triage, and diagnostic tests	Community-based strategies	Facility-based strategies	Strategies for increasing awareness and demand
Physical form and appearance Sample type (including potential discomfort and stigma associated) Accuracy (including false positives and false negatives and their downstream effects) Likelihood for inconclusive result Trust in test results Need for second confirmatory test Test processing time Location where test can be performed Ability for self-testing Test costs (direct)	Proximity to home or work Physical infrastructure characteristics Hours and days of week testing are available Need for appointments Wait times Costs for services Characteristics of person delivering services (competence, trustworthiness, language concordance, peer vs. healthcare workers) Perceived privacy/confidentiality (including any stigma-related concerns) Perceived gender or cultural appropriateness Perceptions/recommendations of loved ones and trusted friends and colleagues Availability of other health and non-health services Turnaround time for test results Pre-test education How results are communicated (e.g., SMS, phone call, in-person) Post-test education and support Enablers/incentives (transport reimbursement, financial)		Most preferred and trusted channels (written, visual, digital, oral; formal vs. informal) Most preferred and trusted messengers (healthcare workers, community leaders, religious leaders, family members, friends, TB survivors) Most resonant and appealing messages, including those that address TB stigma (differing content and framing) Most resonant and appealing design (colors, images, font, layout)
	Location within the community Continuous or event-based availability and frequency Availability and type of linkage support if screening/diagnostic tests are positive	Prioritizing which facilities receive “enhanced” services	
**Combined features**: *How does the combination of different features affect preference and choice?*			
**Trade-offs**: *What trade-offs are acceptable (including potential benefits and harms) and what would people be willing to trade to have their most preferred features included?*			
**Preference heterogeneity**: *How do perspectives, values, and preferences differ across groups and settings as well as by latent class group?*			
